# Social Dominance Orientation Boosts Collective Action Among Low-Status Groups

**DOI:** 10.3389/fpsyg.2021.681302

**Published:** 2021-06-11

**Authors:** Catarina L. Carvalho, Isabel R. Pinto, Rui Costa-Lopes, Darío Páez, Mariana P. Miranda, José M. Marques

**Affiliations:** ^1^Faculty of Psychology and Education Sciences, University of Porto, Porto, Portugal; ^2^Institute of Social Sciences, University of Lisbon, Lisbon, Portugal; ^3^Faculty of Psychology, University of the Basque Country, San Sebastian-Donostia, Spain

**Keywords:** social dominance orientation, social competition, collective action, unstable social hierarchies, social identity theory

## Abstract

We propose that low-status group members' support for group-based hierarchy and inequality (i.e., social dominance orientation; SDO) may represent an ideological strategy to guarantee the legitimacy of future ingroup status-enhancement. Specifically, we argue that, under unstable social structure conditions, SDO serves as an ideological justification for collective action tendencies aimed at competing for a higher status. In such context, SDO should be positively related with actions aimed to favor the ingroup (i.e., collective actions) by increasing group members' motivation to engage in direct competition with a relevant higher-status outgroup. We conducted two studies under highly competitive and unstable social structure contexts using real life groups. In Study 1 (*N* = 77), we induced Low vs. High Ingroup (University) Status and in Study 2 (*N* = 220) we used competing sports groups. Overall, results showed that, among members of low-status groups, SDO consistently increased individuals' motivation to get involved in actions favoring the ingroup, by boosting their motivation to compete with the opposing high-status outgroup. We discuss the results in light of the social dominance and collective action framework.

## Introduction

Members of low-status groups often engage in actions aimed to improve their position in the existing hierarchical social system. Although these actions are more frequently motivated by concerns about equality of treatment, opportunities, and rights for all social groups (e.g., civil rights movements), they can also be motivated by a desire to achieve (or based on beliefs that the ingroup deserves and can reach) more power, privilege and resources than relevant outgroups (see Tajfel, 1978; Tajfel and Turner, 1979; Rubin et al., 2014). In this case, members of low-status groups should feel motivated to compete for social status with a relevant high-status outgroup, and to affirm ingroup's superiority, achieve a positive ingroup distinctiveness and ensure intergroup differentiation (social identity theory, SIT; Tajfel, 1978; Tajfel and Turner, 1979). This is the case in competition-based intergroup contexts like sports, University rankings, political elections, countries (e.g., competing for economic or technological dominance, such as the case of USA vs. China), or even regions or sub-states (e.g., pro-independence movements such as the case of India independence movement from 1857 to 1947 or the ongoing Catalonia pro-independence movements). These competition-based intergroup contexts are framed in hierarchically structured intergroup relations that have to remain in order to be possible for the low-status group to reach and to hold a superior position relative to the relevant outgroup in the future. Actions on behalf of ingroup's interests to achieve the higher status position in the future represent a social competition strategy (e.g., Tajfel, 1978; Tajfel and Turner, 1979). For members of low-status groups to feel motivated to favor the ingroup and engage in social competition, through collective actions, and to attempt to move their group to the top of the status hierarchy, they have to strongly identify with their group and to perceive group boundaries as impermeable, intergroup status positions as illegitimate or unfair and susceptible to change (i.e., unstable intergroup relations; e.g., Tajfel, 1978). For instance, Mummendey et al. (1999) found evidence of a positive relationship between the perception that intergroup relations were unstable, illegitimate, and impermeable, and an increased in social competition in the context of divided Germany. Indeed, under such sociostructural contexts, social competition is the most typical strategy aimed at change status relations between groups (e.g., Blanz et al., 1998; Mummendey et al., 1999). Moreover, these are the contexts that should generate intergroup conflict, in that competition aimed at reversing ingroup and outgroup status relations implies that group members are especially motivated to favor the ingroup (ingroup favoritism) and derogate the outgroup (e.g., Tajfel, 1978).

Therefore, ingroup status-enhancement motivation should, thus, be anchored in hierarchy-enhancing ideologies (i.e., social dominance orientation; SDO; e.g., Sidanius et al., 1994), to guarantee the legitimacy of future ingroup high-status by supporting the existing hierarchically structured social system (see Owuamalam et al., 2016).

### Support for Group-Based Hierarchies and Inequality

According to social dominance theory (SDT; e.g., Sidanius et al., 1994), SDO represents the “desire to establish and maintain hierarchically structured intergroup relations regardless of the position of one's own group(s) within this hierarchy” (Sidanius et al., 2017, p. 152) and the “extent to which one desires that one's ingroup dominate and be superior to out-groups” (Pratto et al., 1994, p. 742). SDT also predicts that members of high-status groups tend to support more strongly group-based hierarchy and inequality (i.e., displaying higher SDO) than members of low-status groups (e.g., Sidanius et al., 1994; Levin, 2004). Indeed, group-based equality is inconsistent with privileged groups' interests as equality would decrease their group's status and power, while promoting the status and increasing the power of disadvantaged groups (e.g., Schmitt et al., 2003). Thus, SDO endorsement by members of low-status groups is believed to be associated with negative feelings about group membership, or to fulfill a palliative function helping these members to cope with cognitive dissonance, anxiety, discomfort and uncertainty resulting from their disadvantaged condition, and leading them to engage in intense justifications or rationalizations of the *status quo* (e.g., Levin and Sidanius, 1999; Jost et al., 2004). According to this view SDO is expected to be negatively associated with willingness to engage in collective actions, toward social change, among members of low-status groups (e.g., Jost et al., 2012, 2017; Osborne et al., 2018).

However, recent evidence has shown that members of low-status groups may also support hierarchical social systems to favor the ingroup. For instance, Brandt and Reyna (2017) observed that people may be, simultaneously, in favor of social change (i.e., improve ingroup low-status) and supportive of inequality (i.e., maintaining the hierarchical system and groups differentiation). Caricati and Sollami's work (Caricati and Sollami, 2017, 2018) on the mechanisms of legitimization of hierarchical social systems, suggests that members of low-status groups may justify and legitimate the social hierarchy if they perceive that they can take some advantages from it and protect ingroup interests.

In line with the above idea, Owuamalam et al. [e.g,. 2016; 2018] proposes the social identity model of system attitudes (SIMSA) suggesting that the support for hierarchical social systems among members of low-status groups can be explained by social identity motives. It is argued that support for such social systems, may actually be a way to maintain a positive social identity (i.e., to satisfy their social identity needs) and a strategy on behalf of ingroup's interests and goals. Specifically, and more important to our research, it is proposed that low-status groups may support hierarchical social systems that, at a first glance, seem to disadvantage their group, because they believe they can benefit from such system in the future (Owuamalam et al., 2016, 2017, 2018, 2019). This *hope for the future ingroup high-status* motive to support hierarchical systems (e.g., Owuamalam et al., 2018) may lead members of low-status groups to support hierarchy-enhancing ideologies, such as SDO, as a mean to guarantee the legitimacy of future ingroup status-enhancement. In other words, members of low-status groups may support hierarchical intergroup relations, intergroup inequality, and status differentials (i.e., SDO), because the only possibility for their group to guarantee and legitimate future high-status is through the maintenance of these unequal hierarchical social systems. Of course, this should only occur if the status hierarchy is perceived to be unstable, thus, susceptible to change in the future (e.g., Tajfel, 1978; Owuamalam et al., 2017, 2018).

#### The Context-Dependent Nature of SDO

In line with the above idea, and in spite of the fact that SDO has been most often conceptualized and operationalized as a relatively stable individual general orientation toward intergroup inequality (e.g., Sidanius et al., 1994), recent evidence has shown that SDO may, in fact, be context-dependent. For instance, SDO levels were found to be shaped by group membership and degree of ingroup identification, sensitive to social competition, to social influence processes, to ingroup status, group dynamics, expected power, and to perceptions of threat (e.g., Reynolds et al., 2001; Guimond et al., 2003; Schmitt et al., 2003; Lehmiller and Schmitt, 2007; Liu et al., 2008; Duckitt and Sibley, 2009; Morrison et al., 2009; Jetten and Iyer, 2010). Specifically, and more relevant to our research, Duckitt and Sibley (2009) highlight that SDO emerges from a competitive worldview developed in contexts of group dominance, inequality and competition, Thus, when social competition increases (e.g., under the shape of struggle for resources, status and power) levels of SDO are expected to increase across all social groups (Sibley and Wilson, 2007; Duckitt and Sibley, 2009; Perry et al., 2013). As Duckitt and Sibley (2009, p. 106, emphasis added) noted, SDO “expresses the competitively driven motivation to *maintain* or *establish* group dominance and superiority.” Thus, in a social structure that favors social competition, high-status groups should feel motivated to protect and *maintain* their superior position and low-status groups should feel motivated to prove and to *establish* their superiority too.

#### The Two Dimensions of SDO

Additionally, the SDO scale was initially conceptualized and designed in terms of a single dimension (Pratto et al., 1994). However, evidence has shown that this construct should be conceptualized and operationalized as having two distinct dimensions, reflecting each one distinct psychological orientation (Jost and Thompson, 2000; Kugler et al., 2010; Ho et al., 2015). One such dimension reflects support for group-based dominance hierarchies (SDO-D), defined as the support for hierarchical social systems “in which dominant groups actively oppress subordinate groups [and] will be related to phenomena such as support for aggressive intergroup behavior, support of overtly negative intergroup attitudes, support for negative allocations to outgroups, and the perception of group-based competition” (Ho et al., 2012, p. 585). The other dimension reflects opposition to group-based equality (SDO-E), defined as “an aversion to the general principle of equality and to reducing the level of hierarchy between social groups. Opposition to equality translates psychologically into support for exclusivity” (Ho et al., 2012, p. 585). Thus, according to Ho et al. (2012, 2015), examining these two subdimensions separately allows to better understand, and more accurately predict, intergroup attitudes and behaviors. Indeed, and relevant for our research, Jost and Thompson (2000) suggested that using SDO as a unidimensional construct may fail to assess ideological processes among members of low-status groups (see also Kugler et al., 2010).

### The Present Research

With the present research, we attempted to combine the above-mentioned contributions of SIT and SDT and the novel *hope for the future ingroup high-status* explanation to support hierarchical systems (SIMSA; e.g., Owuamalam et al., 2018, 2019). We argue that support for group-based hierarchies and inequality (i.e., SDO) stand for an ideological strategy aimed to guarantee the legitimacy of future ingroup status-enhancement among members of low-status groups, namely in unstable social structures (the type of social structure believed to lead low-status group members to engage in social competition, according to SIT). Specifically, SDO endorsement among members of low-status groups may represent a strategy intended to maintain the existing hierarchical social system (i.e., maintenance of hierarchical organized intergroup relations and group status differentials), in order to ensure a legitimate future advancement of the ingroup within the prevailing status hierarchy. In this case, SDO should be positively associated with actions aimed to improve ingroup conditions, status, power and influence, to overcome the high-status outgroup, and to achieve positive ingroup distinctiveness (i.e., collective actions; e.g., Tajfel, 1978). Thus, in competitive social structure conditions, SDO should boost collective action tendencies among members of low-status groups, as a means to favor the ingroup by increasing the motivation to engage in direct competition with relevant high-status outgroups. This would reflect an ideological strategy aimed to guarantee the legitimacy of future ingroup high-status. Among members of high-status groups, although they may also feel motivated to compete for the maintenance of the higher status, we expect different patterns of association.

Moreover, although the literature suggests that SDO is positively associated with ingroup favoritism only in high-status groups (e.g., Levin et al., 2002), we propose that in competition-based intergroup contexts, a positive relationship may exist between SDO and ingroup favoritism (and outgroup derogation), among members of low-status groups.

In order to test the above ideas, we conducted two studies under unstable social structure contexts. In Study 1 we induced Low vs. High Ingroup Status based on information from a University ranking, and, in Study 2, we used football team supporters from two opposing teams with huge rivalry between them, during a football championship.

According to SIT's predictions, given the highly unstable social structure contexts (i.e., the necessary conditions for social competition strategies to emerge), we expect that, in both studies, members of low-status groups (as compared to members of high-status groups) show higher beliefs that status positions between groups are illegitimate and unstable; report stronger motivation to favor the ingroup and derogate the outgroup; and show stronger social competition intentions (H1). We also expect members of both low- and high-status groups to be equally and strongly identified with their group.

Moreover, assuming that SDO represents an ingroup status-enhancement strategy to guarantee the legitimacy of future ingroup high-status, we expect SDO to be positively related to social competition intentions and collective action tendencies among low-status groups (H2). As a result, we should observe positive associations between SDO and ingroup favoritism and/or outgroup derogation, among low-status groups. Among high-status groups, these positive associations are already expected according to previous research (e.g., Levin and Sidanius, 1999).

Finally, we expect to find, among members of the low-status groups, a mediational process in which SDO boosts collective action tendencies to favor the ingroup by increasing individuals' motivation to engage in direct competition with the other relevant high-status outgroup (H3). In other words, we expect social competition intentions to explain the relation between SDO and collective action tendencies, reflecting the proposed function of SDO as a strategy to guarantee the legitimacy of future ingroup high-status.

## Study 1

### Materials and Method

#### Participants and Design

Participants were 77 students enrolled at the University of Porto (convenience sample; 43 female and 34 male), aged between 18 and 42 (*M* = 21.01, *SD* = 3.82), who were randomly assigned to one of the two Ingroup Status conditions (Ingroup Status: Low vs. High-Status).

Participants' sex and age did not significantly differ across conditions, respectively, χ^2^(1) = 0.13, *p* = 0.820, and *t*(75) = 0.39, *p* = 0.701.

#### Procedure

Participants were contacted in the street and were invited to fill out an opinion survey about the Quality of Education and Employability in the University of Porto.

Participation was voluntary and not monetarily compensated. After giving informed consent, participants provided demographic information (e.g., age, sex). Then, to induce Low vs. High-status beliefs about University of Porto, they read, at the beginning of the questionnaire, one of two newspaper headlines about the results of the annual QS World University Ranking, (1) High-Status condition: “The University of Porto is the best Portuguese University in the QS World University Ranking,” stressing that the University of Porto has maintained its top position over the years, with the University of Lisbon in second place (these are the two Portuguese Universities that always compete for the first place); (2) Low-Status condition: “The University of Lisbon surpasses the University of Porto in the QS World University Ranking,” stressing that the University of Lisbon had dethroned the leadership of the University of Porto in the ranking. Both headlines were genuine but corresponded to results from different years – the headline used in High-Status condition was from 2018 and the headline used in the Low-Status condition was from 2019. The dates of the newspaper headlines were removed.

Upon completion, participants were thanked and fully debriefed about the deceptions involved in the study.

#### Measures

Following the Ingroup Status manipulation, participants reported their beliefs about University of Porto's status (manipulation check), identification with their University, beliefs about the stability and legitimacy of status positions between the two Universities, answered to ingroup favoritism and outgroup derogation measures, and finally completed the SDO, social competition intentions and collective action tendencies' scales.

##### University Status (Manipulation Check)

After reading the headline on the first page, participants indicated their beliefs about the status of the University of Porto in comparison to the University of Lisbon with a single item: “The position held by the University of Porto in the ranking, compared to the University of Lisbon, is ….” (1 = *inferior*; 7 = *superior*).

##### Identification With University of Porto

As a control measure, in order to measure participants' identification with the University of Porto, we used a 4-item scale (based on Pinto et al. (2016); 1 = *I fully disagree*; 7 = *I fully agree*): (1) “In general, I'm proud to belong to the University of Porto.”; (2) “I feel good for being part of the University of Porto.”; (3) “In general, I identify with the University of Porto.”; (4) “I have a strong connection with the University of Porto.”. A principal components factorial analysis conducted on these items extracted one single factor accounting for 69% of the total variance. We averaged the scores of the items to an *identification* index (Cronbach's α = 0.85).

##### Stability of Ranking Positions

We measured participants' beliefs about the stability of status positions between the University of Porto and the University of Lisbon with two items (based on Owuamalam et al. (2016); 1 = *I fully disagree*; 7 = *I fully agree*): (1) “I believe that the positions of these two Universities in the ranking will remain the same in the future.”; (2) “I believe that the positions of these two Universities in the ranking can reverse in the future.” (reversed-coded). We averaged the scores of the items to a *stability* index (Pearson product-moment correlations between items: *r* = 0.38, *p* ≤ 0.001), such that higher scores represented beliefs in stable ranking positions.

##### Legitimacy of Ranking Positions

We also asked participants about the legitimacy of the positions of the two Universities in the ranking (1 = *I fully disagree*; 7 = *I fully agree*): (1) “I believe that the positions of these two Universities, in the ranking, is legitimate.”; (2) “I believe that the positions of these two Universities, in the ranking, is fair.”. We averaged the scores of the items to a *legitimacy* index (Pearson product-moment correlations between items: *r* =0.60, *p* ≤ 0.001), such that higher scores represent beliefs in legitimate ranking positions.

##### Ingroup Favoritism and Outgroup Derogation

We also included ingroup favoritism and outgroup derogation measures. We are especially interested to observe the relationships of these measures with our main variable (SDO) among participants in the low-status condition. We measured participants' favoritism toward their group and outgroup derogation with four items (1 = *I fully disagree*; 7 = *I fully agree*): (1) “Regardless of the rankings, the University of Porto is the best University.”; (2) “Regardless of the rankings, the University of Porto is the best place to study.”; (3) “I do not sympathize with the University of Lisbon.”; (4) “The University of Lisbon has more fame than quality.”. A principal components factorial analysis conducted on these items extracted two factors accounting for 83% of the total variance. We averaged the scores of items 1 and 2 to an *ingroup favoritism* index (Pearson product-moment correlations between items: *r* = 0.82, *p* ≤ 0.001), and items 3 and 4 to an *outgroup derogation* index (Pearson product-moment correlations between items: *r* = 0.49, *p* ≤ 0.001).

##### SDO

Participants responded to the full 16*-*item SDO_7_ scale (Ho et al., 2015)^1^, on 7-point scales (1 = *I fully disagree*; 7 = *I fully agree*). A confirmatory factor analysis showed that the two-dimensional solution had an acceptable model fit. We averaged the scores of the items and composed the SDO-D and SDO-E subdimensions according to Ho et al.'s (2015) theoretical framework and guidelines.

Both SDO-D (e.g., “An ideal society requires some groups to be on top and others to be on the bottom.”; Cronbach's α = 0.75) and SDO-E dimensions (e.g., “It is unjust to try to make groups equal.”; Cronbach's α = 0.88) were reliable.

##### Social Competition

To measure participants' motivation to compete with the outgroup (University of Lisbon), we use a 4-item scale adapted to our context (based on Blanz et al. (1998); 1 = *I fully disagree*; 7 = *I fully agree*): (1) “We are going to make it clear to everyone that the students of the University of Porto are more efficient than the students of the University of Lisbon.”; (2) “We will show very soon that the students of the University of Porto have more initiative and commitment than the students of the University of Lisbon.”; (3) “We, the students of the University of Porto, have to work harder to have a higher academic reputation than the students of the University of Lisbon.”; (4) “We, the students of the University of Porto, should strive to achieve greater success than the students of the University of Lisbon.”. A principal components factorial analysis conducted on these items extracted one single factor accounting for 79% of the total variance. We averaged the scores of the items to a *social competition* index (Cronbach's α = 0.91).

##### Collective Action

Finally, we asked participants to indicate their motivation to participate in 6 collective initiatives in favor of the University of Porto (1 = *not motivated al all*, 7 = *very motivated*): (1) “Participate in a meeting/discussion to define strategies to increase the University's potential.”; (2) “Participate in a meeting/discussion to define strategies so that the University of Porto achieves more success.”; (3) “Act together with other students to defend University's interests.”; (4) “Act together with other students to defend University's image.”; (5) “Act together with other students to increase University's prestige.”, (6) “Act together with other students to increase University's status.”. A principal components factorial analysis conducted on these items extracted one single factor accounting for 73% of the total variance. Thus, we averaged the scores to a *collective action* index (Cronbach's α = 0.93)^2^.

### Results

Table 1 summarizes the descriptive statistics and correlations between measures, by ingroup status condition.

**Table 1:** Summary of means, standard deviations, and correlations between measures by ingroup status condition.

		**Low-statuscondition**		**High-status condition**											
	**Variable**	***M***	***SD***	***M***	***SD***	**1**	**2**	**3**	**4**	**5**	**6**	**7**	**8**	**9**	**10**
1	University status	4.79	1.52	5.68	1.40		0.38^*^	−0.06	0.31^†^	0.55^***^	−0.02	0.25	−0.15	0.09	0.10
2	Identification	5.74	0.97	5.93	0.97	0.31^†^		0.04	0.41^**^	0.26	−0.31^†^	0.20	−0.15	0.19	0.08
3	Stability	3.00	1.19	4.16	1.21	−0.25	−0.17		0.27	0.04	0.06	0.05	0.27	−0.06	−0.11
4	Legitimacy	4.55	1.24	5.52	1.18	0.11	−0.19	0.30^†^		0.40^*^	0.17	0.55^***^	0.19	0.47^**^	0.33^*^
5	Ingroup favoritism	5.50	1.47	4.91	1.66	0.42^**^	0.70^***^	−0.24	−0.25		0.29^†^	0.35^*^	−0.16	0.37^*^	0.16
6	Outgroup derogation	3.87	1.37	2.70	1.24	0.14	0.04	−0.08	−0.30^†^	0.12		0.21	31^†^	0.35^*^	0.02
7	SDO-D	2.93	1.29	3.07	0.85	0.38^*^	0.18	0.06	−0.13	0.19	0.24		0.51^***^	0.51^***^	0.24
8	SDO-E	2.39	1.27	2.61	1.09	0.44^**^	0.14	−0.12	−0.17	0.17	0.31^†^	0.74^***^		0.21	0.00
9	Social competition	4.04	1.83	3.24	1.86	0.49^***^	0.36^*^	0.00	−0.39^*^	0.49^***^	0.30^†^	0.64^***^	0.54^***^		0.32^*^
10	Collective action	4.32	1.78	4.21	1.63	0.30^†^	0.50^***^	−0.20	−0.32^†^	0.55^***^	0.15	0.40^*^	0.44^**^	0.59^***^	

† *p ≤ 0.10*;

* *p ≤ 0.05*;

** *p ≤ 0.01*;

*** *p ≤ 0.001*.

*Correlations for the Low-status condition (n = 39) are below the diagonal; and correlations for High-status condition (n = 38) are above the diagonal*.

Results show significant differences in participants' beliefs about University status, those being higher in the High-Status than in the Low-Status condition, *t*(75) = 2.68, *p* = 0.009, Cohen's *d* = 0.62. This result shows that ingroup status manipulation was effective to induce lower vs. higher beliefs about university status (see Table 1).

As expected, there are no significant differences between conditions regarding participants' ingroup identification, *t*(75) = 0.84, *p* = 0.401, and participants in both conditions are strongly identified with their group (>5.50, on a 7-point rating-scale). As predicted, participants in the Low-Status condition show lower beliefs about stability, *t*(75) = 4.23, *p* ≤ 0.001, Cohen's *d* = 0.98, and legitimacy, *t*(75) = 3.49, *p* ≤ 0.001, Cohen's *d* = 0.81, than participants in the High-Status condition (i.e., higher beliefs in illegitimate and unstable status positions between groups, the necessary conditions for social competition to emerge among members of low-status groups).

Results also show that participants in the Low-Status condition show stronger outgroup derogation, *t*(75) = 3.91, *p* ≤ 0.001, Cohen's *d* = 0.90, than participants in the High-Status condition (see Table 1). There are no significant differences between conditions in the remaining measures.

We also expected SDO to be positively related to social competition intentions and collective action tendencies. Thus, by observing the Pearson product-moment correlations between all measures (see Table 1) we observe that, among participants in the Low-Status condition, both SDO-D and SDO-E are positively related with social competition (*r* = 0.64, *p* ≤ 0.001, and *r* =0.54, *p* ≤ 0.001, respectively) and collective action (*r* =0.40, *p* = 011, and *r* =0.44, *p* = 0.005, respectively). We also observe a positive association only between SDO-E and outgroup derogation, although not reaching statistical significance (*r* = 0.31, *p* = 0.054).

Among participants in the High-Status condition, we observe that only SDO-D is positively related with social competition (*r* = 0.51, *p* ≤ 0.001), but not SDO-E (*r* = 0.21, *p* = 0.207); neither SDO-D or SDO-E are related to collective action (*r* = 0.24, *p* = 0.145; *r* = 0.00, *p* = 0.986, respectively).

#### SDO as an Ideological Strategy to Legitimate Future Ingroup Status-Enhancement

We expected that SDO should be positively associated with collective action tendencies by increasing social competition intentions (i.e., motivation to engage in direct competition with the opposing outgroup), among members of the low-status groups.

To test the effect of SDO on collective action tendencies through social competition intentions, we conducted a mediation analysis (using PROCESS 3.5 version, Model 4 with 1,000 bootstrap samples; Hayes, 2018), considering SDO as the predictor, social competition as the mediator, and collective action as the dependent measure^3^. Specifically, we tested four independent mediation models: two independent models for each SDO subdimensions, for each status condition (two for the low-status condition and two for the high-status condition). All reported coefficients are unstandardized.

**Figure 1:**
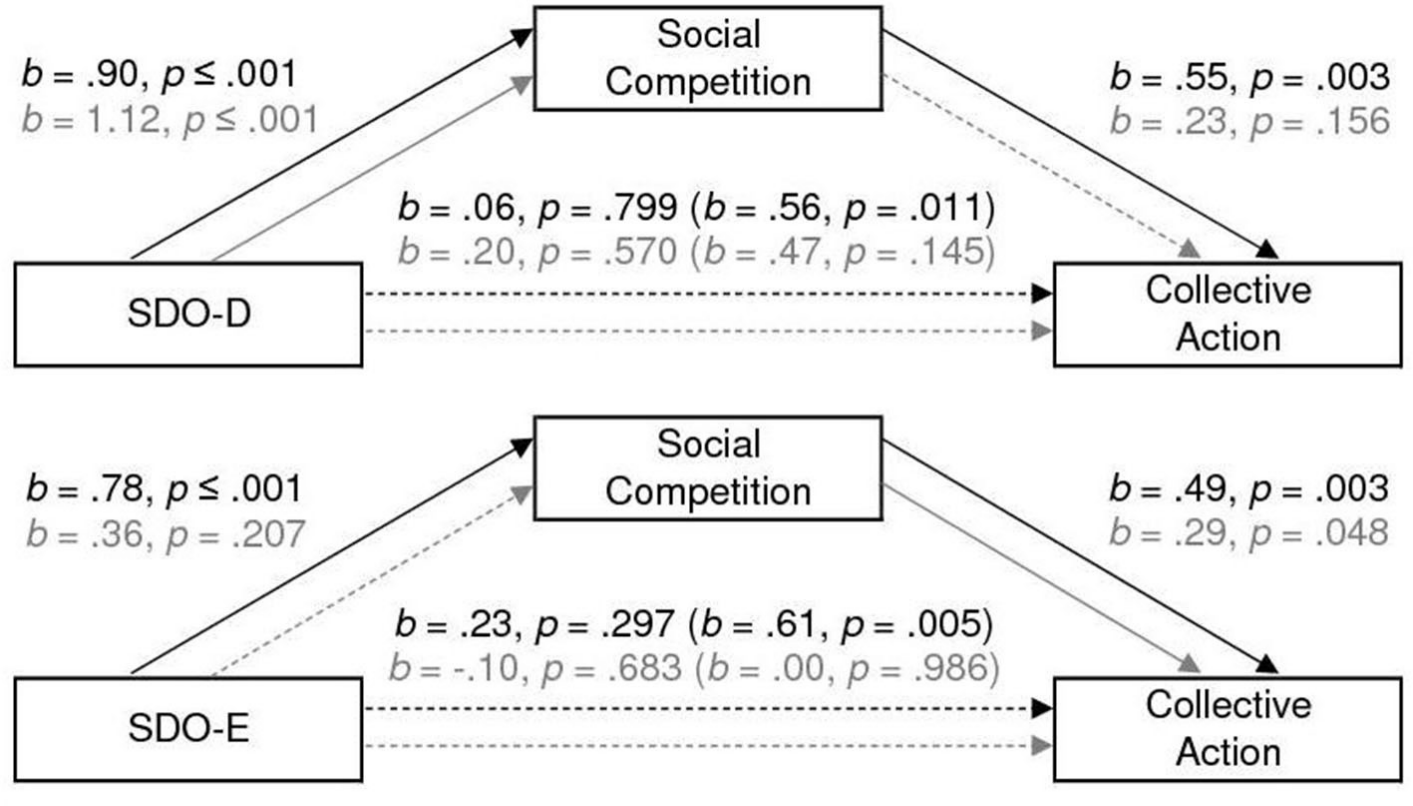
The effect of SDO on collective action mediated by social competition. Coefficients for the high-status condition are in gray. All reported coefficients are unstandardized.

As we can see in Figure 1, SDO-D is a significant predictor of social competition in both Low- and High-status conditions, however, social competition predicts collective action only in the Low-status condition. The model explains 35% of the variability observed in collective action, *F*_(2,36)_ = 9.80, *p* ≤ 0.001, in the Low-status condition; and 11% of the variability observed in collective action, *F*_(2,35)_ = 2.20, *p* = 0.126, in the High-status condition. The indirect effect of SDO-D on collective Action through social competition is positive and significant in the Low-status condition, *b* = 0.50, *SE* = 0.18, 95% CI [0.167, 0.842], a *post-hoc* power analysis showed a power (1 – β)^4^ of 0.88, and non-significant in the High-status condition, *b* = 0.26, *SE* = 0.22, 95% CI [−0.201, 0.719], power (1 – β) of 0.28.

We observe (see Figure 1) that SDO-E is also a significant predictor of social competition only in the Low-status conditions; social competition predicts collective action at both Low- and High-status conditions. The model explains 37% of the variability observed in collective action, *F*_(2,36)_ = 10.61, *p* ≤ 0.001, in the Low-status condition; and 11% of the variability observed in collective action, *F*_(2,35)_ = 2.11, *p* = 0.137, in the High-status condition. The indirect effect of SDO-E on collective action through social competition is positive and significant in the Low-status condition, *b* = 0.38, *SE* = 0.16, 95% CI [0.102, 0.732], a *post-hoc* power analysis showed a power (1 – β) 0.84, and non-significant in the High-status condition, *b* = 0.11, *SE* = 0.10, 95% CI [−0.065, 0.339], power (1 – β) of 0.12.

Results support the mediational hypothesis (full mediation) for both the SDO-D and SDO-E dimension only among participants in the Low-Status condition.

### Discussion

Overall, results showed that, among participants in the Low-status condition, social competition fully mediates the relationship between SDO (both SDO-D and SDO-E) and collective action, suggesting that SDO (shaped by the specific competition-based context, highly unstable) boosted individuals' motivation to compete with the other relevant high-status outgroup, and in turn, increased their motivation to get involved in actions to favor the ingroup and to contest ingroup position in the ranking (based on results with contestation measure; see OSM 1 and 2).

On the contrary, among participants in the High-Status condition, we found different patterns of associations, and SDO did not increase collective action tendencies, possibly because they believe to have a secure high-status.

## Study 2

In Study 1 we induced (Low vs. High) ingroup status in a highly unstable social structure context. As expected, we found that, in the Low-status condition, participants' SDO boosted collective action tendencies by increasing their motivation to engage in direct competition with the opposing high-status group.

In Study 2, we intended to test our predictions with another type of group whose existence is based on, and is inherent to, highly competitive and unstable social structure context. Thus, in Study 2 we used football team supporters of two opposing teams whose relations are marked by huge rivalry, during a football championship.

Since the football context is often associated to violent events among teams' supporters, in Study 2 we test our model predicting both normative (i.e., actions supporting the ingroup, consistent with ingroup favoritism) and non-normative collective action (i.e., actions harming the outgroup, consistent with outgroup derogation). Moreover, since SDO has been found to be positively related to aggressive intergroup attitudes and behaviors against outgroups (e.g., Ho et al., 2012), we may expect stronger associations with outgroup derogation (than with ingroup favoritism) and non-normative collective action (than with normative collective actions).

### Materials and Method

#### Participants

Participants were 220 supporters (convenience sample; 107 female and 113 male) of two of the major Portuguese football teams (119 from the *Futebol Clube do Porto* and 101 from *Sport Lisboa e Benfica*), aged between 18 and 61 (*M* = 25.92, *SD* = 7.94).

Participants' sex and age did not significantly differ across team samples, respectively, **χ**^**2**^(1) = 2.75, *p* = 0.106, and *t*(218) = 0.24, *p* = 0.810.

#### Procedure

Participants were contacted through Facebook groups from both football teams to fill out a survey about the regular occurrence of some extreme interactions between football fans, leading from time to time to violent events. The study was conducted during the championship of 2019/2020, in the months of February and March. During that period, the team leading the ranking scores (the team which occupied the first place in the ranking at that time; i.e., high-status group) was *Sport Lisboa e Benfica* (SLB) followed by the *Futebol Clube do Porto* (FCP) (the team occupying the second position in the ranking; i.e., low-status group). SLB was also the team that had won the previous championship of 2018/2019, and in 2017/2018 the winner had been the FCP. Thus, the championship is a very unstable context, and either team could win the 2019/2020 championship. To highlight this unstable context, at the beginning of the questionnaire it was stressed the fact that in the last 18 years, the title of national football champion (Portuguese Cup) had been awarded only to these two football teams (real information).

Participation was completely voluntary and not monetarily compensated. After giving informed consent, participants provided demographic information (e.g., age, sex). Upon completion, participants were thanked, and the aim of the study was clarified.

#### Measures

Firstly, after selecting their favorite team from a list of all national football teams (those who selected other teams, were redirected to the end of the questionnaire), as a control measure, participants indicated identification with their football team. Then, participants reported their beliefs about teams' status, about the stability and legitimacy of ranking positions between the two teams (that ultimately reflects teams' status), answered to ingroup favoritism and outgroup derogation measures, and finally, completed the SDO, social competition intentions and collective action tendencies scales. As mentioned above, in this study we included normative collective actions supporting their own team (i.e., actions to favor the ingroup) and non-normative violent collective actions against the opposing team (i.e., actions to harm and derogate the outgroup), since that last type of actions is very common to occur in the context of football and moreover, it was, supposedly, the aim of the study (cover story).

##### Identification With the Team

As a control measure, we assessed participants' identification with their team using four items from Leach et al. (2008)' scale (1 = *I fully disagree*; 7 = *I fully agree*): (1) “I am glad to be a supporter of the FCP/SLB.”; (2) “I think that the FCP/SLB' supporters have a lot to be proud of.”; (3) “It is pleasant to be a supporter of the FCP/SLB.”; (4) “Being a supporter of the FCP/SLB gives me a good feeling.”. A principal components factorial analysis conducted on these items extracted one single factor accounting for 77% of the total variance. We averaged the scores of the items to an *identification* index (Cronbach's α = 0.90).

##### Team Status

Participants indicated their beliefs about their team's status in comparison to the opposing team: “The status of FCP/SLB, compared to the SLB/FCP, is ….” (1 = *inferior*; 7 = *superior*).

##### Stability of Ranking Positions

We measured participants' beliefs about the stability of ranking positions between the two teams with two items (based on Owuamalam et al. (2016); 1 = *I fully disagree*; 7 = *I fully agree*): (1) “The positions of these two teams in the ranking, can easily reverse in the future.” (reversed-coded); (2) “The team that currently leads the ranking, can easily lower its position in the future.” (reversed-coded). We averaged the scores of the items to a *stability* index (Pearson product-moment correlations between items: *r* = 0.50, *p* ≤ 0.001), such that higher scores represent perceived stable ranking positions.

##### Legitimacy of Ranking Positions

We also asked participants about the legitimacy of the positions of these two teams (1 = *I fully disagree*; 7 = *I fully agree*): “The positions of these two teams in the ranking … (1) is legitimate.”; (2) “is unfair.” (reversed-coded). We averaged the scores of the items to a *legitimacy* index (Pearson product-moment correlations between items: *r* =0.63, *p* ≤ 0.001), such that higher scores represented beliefs in legitimate ranking positions.

##### Ingroup Favoritism and Outgroup Derogation

We measured participants' motivation to favor their team and motivation to derogate the opposing team in a 4-items scale (1 = *I fully disagree*; 7 = *I fully agree*): (1) “Whether they win or lose, my team is the best team.”; (2) “Whether they win or lose, my team is the best I could belong to.”; (3) “The SLB [vs. FCP outgroup] has more fame than value.”; (4) “The SLB [vs. FCP outgroup] plays very poorly.”. A principal components factorial analysis conducted on these items extracted two factors accounting for 79% of the total variance. We averaged the scores of the items 1 and 2 to an *ingroup favoritism* index (Pearson product-moment correlations between items: *r* =0.64, *p* ≤ 0.001), and the items 3 and 4 to an *outgroup derogation* index (Pearson product-moment correlations between items: *r* =0.53, *p* ≤ 0.001).

##### SDO

Participants answered to the full 16*-*item SDO_7_ scale (Ho et al., 2015), on 7-point scales (1 = *I fully disagree*; 7 = *I fully agree*). A confirmatory factor analysis showed that the two-dimensional solution had an acceptable model fit. We averaged the scores of the items and composed the SDO-D and SDO-E subdimensions according to Ho et al.'s (2015) theoretical framework and guidelines. Both SDO-D (Cronbach's α = 0.78) and SDO-E dimensions (Cronbach's α = 0.86) were reliable.

##### Social Competition

To measure participants' motivation to compete with the outgroup, we used a 4-item scale adapted to our context (based on Blanz et al. (1998); 1 = *I fully disagree*; 7 = *I fully agree*): (1) “The FCP (vs. SLB) is going to make it clear to everyone that it is more efficient than the SLB (vs. FCP).”; (2) “The FCP (vs. SLB) has to work more to have a higher international reputation than the SLB (vs. FCP).”; (3) “The FCP (vs. SLB) is going to show everyone that it is more offensive and attacking team than the SLB (vs. FCP).”; (4) “The FCP (vs. SLB) is going to teach the SLB (vs. FCP) how to play football.”. A principal components factorial analysis conducted on these items extracted one single factor accounting for 80% of the total variance^5^. We averaged the scores of the items to a *social competition* index (Cronbach's α = 0.87).

##### Collective Action

Finally, we asked participants to indicate their motivation to participate, in the future, in (normative and non-normative) collective initiatives, either supporting their team or harming the rival team (1 = *not motivated al all*, 7 = *very motivated*): “Join other supporters of my team … (1) and wait for the team bus to congratulate the team when they win.”; (2) wait for the team bus to show my support for the team, even when they lose.”; (3) to block the rival team bus.”; (4) to invade and damage the headquarters of opposing team.”; (5) to shoot petards at supporters of rival team.”; (6) to paint walls on the street with symbols or slogans alluding to my team.”; (7) set objects or accessories of the rival teams on fire (e.g., scarves, flags, buses).”; (8) to confront supporters from rival team.”. A principal components factorial analysis conducted on these items extracted two factors accounting for 82% of the total variance. We averaged the scores of the items to a *normative CA* index (Pearson product-moment correlations between items: *r* = 0.88, *p* ≤ 0.001) corresponding to items 1 and 2, and a *non-normative CA* index (Cronbach's α = 0.91) corresponding to items 3–8.

### Results

Table 2 summarizes the descriptive statistics and correlations between all measures, by football team.

**Table 2:** Summary of means, standard deviations, and correlations between measures by football team.

		**FCP (Low-status group)**		**SLB (High-status group)**												
	**Variable**	***M***	***SD***	***M***	***SD***	**1**	**2**	**3**	**4**	**5**	**6**	**7**	**8**	**9**	**10**	**11**
1	Identification	6.21	0.98	6.26	0.96		0.51^***^	−0.09	0.29^**^	0.53^***^	0.38^***^	−0.08	−0.11	0.49^***^	0.49^***^	0.27^**^
2	Team status	5.72	1.38	5.98	1.41	0.44^***^		−0.08	0.25^*^	0.60^***^	0.46^***^	0.06	0.03	0.56^***^	0.35^***^	0.23^*^
3	Stability	3.19	1.64	3.64	1.76	−0.14	−0.28^**^		−0.12	−0.19^†^	0.08	0.13	0.11	0.01	0.15	0.12
4	Legitimacy	4.09	1.78	6.17	1.24	−0.24^**^	−0.33^***^	−0.01		0.24^*^	−0.01	0.02	−0.11	0.34^***^	0.17^†^	0.10
5	Ingroup favoritism	6.27	1.10	6.13	1.26	0.60^***^	0.45^***^	−0.27^**^	−0.27^**^		0.36^***^	0.04	−0.00	0.56^***^	0.39^***^	0.17^†^
6	Outgroup derogation	3.81	1.50	3.75	1.81	0.22^*^	0.42^***^	−0.13	−0.34^***^	0.27^**^		0.24^*^	0.23^*^	0.61^***^	0.37^***^	0.40^***^
7	SDO-D	3.08	1.14	3.14	1.23	0.10	0.28^**^	0.03	−0.28^**^	0.12	0.22^*^		0.64^***^	0.19^†^	0.12	0.27^**^
8	SDO-E	2.44	1.15	2.63	1.23	−0.03	0.10	0.03	−0.23^*^	0.08	0.31^***^	0.57^***^		0.15	0.06	0.27^**^
9	Social competition	4.63	1.42	4.93	1.57	0.41^***^	0.52^***^	−0.27^**^	−0.35^***^	0.41^***^	0.43^***^	0.30^***^	0.12		0.51^***^	0.30^**^
10	Normative CA	3.38	2.09	4.04	2.37	0.26^**^	0.41^***^	−0.15	−0.33^***^	0.37^***^	0.28^**^	0.29^***^	0.19^*^	0.41^***^		0.41^***^
11	Non-normative CA	1.44	1.07	1.67	1.39	0.00	0.18^*^	0.15	−0.20^*^	0.04	0.37^***^	0.21^*^	0.33^***^	0.24^*^	0.32^***^	

† *p ≤ 0.10*;

* *p ≤ 0.05*;

** *p ≤ 0.01*;

*** *p ≤ 0.001*.

*Correlations for FCP supporters (n = 119) are below the diagonal; and correlations for SLB supporters (n = 101) are above the diagonal*.

As expected, results showed that supporters from both teams are equally, *t*(218) = 0.38, *p* = 0.708, and strongly identified (>6.20 on a 7-point rating-scale) with their team (see Table 2). There are no significant differences between teams' supporters regarding beliefs about teams' status, *t*(218) = 1.37, *p* = 0.174. This result may be due to the fact that the championship was still ongoing, and the championship winner was not yet established, being possible that either team could win. We may also speculate that this may have occurred as a strategy to elevate the ingroup, in the case of the FCP supporters (low-status group), or based on beliefs that they could still win despite the (real) current lower position in the ranking (i.e., 2nd place).

Consistent with Study 1 and with our predictions, FCP supporters (low-status group) showed lower beliefs about stability (despite not reaching statistical significance), *t*(218) = 1.96, *p* = 0.051, Cohen's *d* = 0.27, and legitimacy, *t*(218) = 9.88, *p* ≤ 0.001, Cohen's *d* = 1.34. Contrary to our predictions there were no differences between teams' supporters regarding ingroup favoritism, *t*(218) = 0.88, *p* = 0.383, outgroup derogation, *t*(218) = 0.262, *p* = 0.793, or social competition intentions, *t*(218) = 1.52, *p* = 0.130. Again, the lack of differences may be because the championship was still ongoing, the winner was not yet established, and both teams' supporters were highly motivated to see their team in the first place.

Finally, we found a higher motivation to get involved in actions supporting their team (Normative CA), *t*(218) = 2.22, *p* = 0.028, Cohen's *d* = 0.33, among the SLB supporters (high-status group), compared to FCP supporters (low-status group; see Table 2). This result may suggest an attempt to reaffirm their current (but not definitive) position.

There are no significant differences between teams' supporters in the remaining measures.

We also expected SDO to be positively related to social competitions intentions and collective action tendencies. Thus, by observing the Pearson product-moment correlations between all measures (see Table 2), we observe that, among FCP supporters (low-status group), only SDO-D (*r* = 0.30, *p* ≤ 0.001) is positively related with social competition, but not SDO-E (*r* = 0.12, *p* = 0.195); both SDO-D (*r* =0.29, *p* ≤ 0.001; *r* =0.21, *p* = 0.025), and SDO-E (*r* = 0.19, *p* = 0.043; *r* = 0.33, *p* ≤ 0.001) are positively related with both normative CA and non-normative CA, respectively. We also observe that both SDO-D (*r* = 0.22, *p* = 0.015) and SDO-E (*r* = 0.31, *p* ≤ 0.001) are positively related only with outgroup derogation.

Among the SLB supporters (high-status group), both SDO-D (*r* = 0.27, *p* = 0.007) and SDO-E (*r* = 0.27, *p* = 0.007) are related with only non-normative CA. We also observe that both SDO-D (*r* = 0.24, *p* = 0.016) and SDO-E (*r* = 0.23, *p* = 0.022) are positively related only with outgroup derogation.

#### SDO as an Ideological Strategy to Legitimate Future Ingroup Status-Enhancement

We expected that SDO would predict collective action tendencies by increasing social competition intentions (i.e., motivation to engage in direct competition with the opposing outgroup).

To test the effect of SDO on collective action tendencies through social competition, we conducted a mediation analysis (using PROCESS 3.5 version, Model 4 with 1,000 bootstrap samples; Hayes, 2018), considering SDO as the predictor, social competition as the mediator, and collective action as the dependent measure. Specifically, we tested eight independent mediation models: four for the low-status group and four for the high-status group, since we have two SDO subdimensions and two types of collective action (normative and non-normative). All reported coefficients are unstandardized.

##### SDO-D on Normative CA Through Social Competition

**Figure 2:**
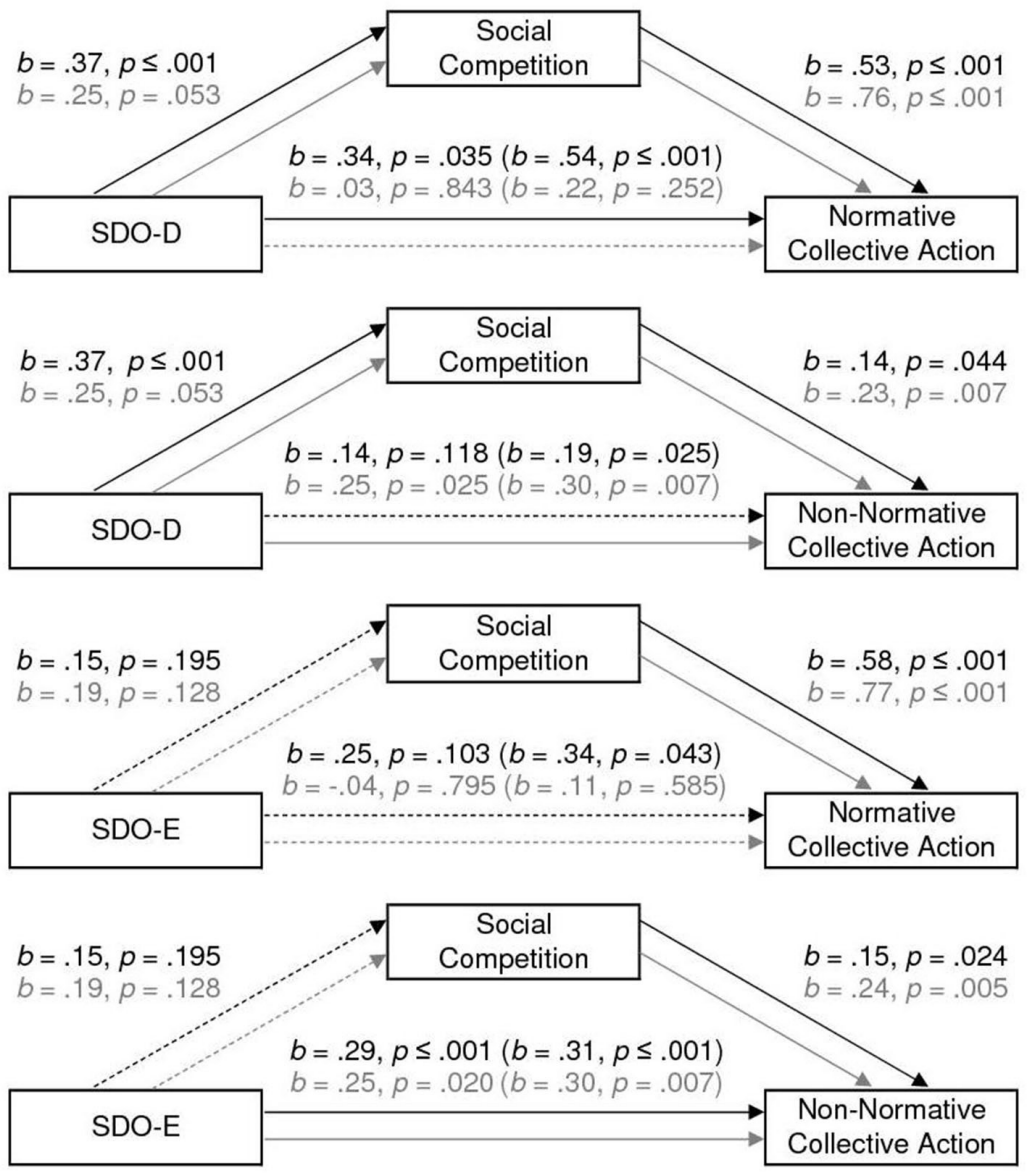
The effect of SDO on normative CA and non-normative CA, mediated by social competition. Coefficients for the SLB supporters (high-status group) are in gray. All reported coefficients are unstandardized.

As we can see in Figure 2, SDO-D is a significant predictor of social competition, and, in turn, social competition predicts normative CA, among both FCP supporters (low-status group) and SLB supporters (high-status group). We also observed that, SDO-D maintains a positive and significant direct effect on normative CA even after the mediator is included, among FCP supporters, *b* = 0.34, *p* = 0.035. The model explains 20% of the variability observed in normative CA, *F*_(2,116)_ = 14.68, *p* ≤ 0.001, among FCP supporters; and 26%, *F*_(2,98)_ = 17.02, *p* ≤ 0.001, among SLB supporters. The indirect effect of SDO-D on normative CA through social competition is positive and significant among FCP supporters, *b* = 0.20 *SE* = 0.07, 95% CI [0.065, 0.344], and a *post-hoc* power analysis showed a power (1 – β) of 0.89; and non-significant among SLB supporters, *b* = 0.19 *SE* = 0.11, 95% CI [−0.006, 0.415], power (1 – β) of 0.50.

##### SDO-D on Non-normative CA Through Social Competition

We also observe a positive direct effect of SDO-D on non-normative CA, among FCP supporters, however, this effect disappear after the mediator is included, *b* = 0.14, *p* = 0.118. We also observed that, SDO-D maintain a positive and significant direct effect on non-normative CA even after the mediator is included, among SLB supporters, *b* = 0.25, *p* = 0.025. Social competition is also a significant predictor of non-normative CA, although weaker, among both FCP supporters and SLB supporters. The model explains 8% of the variability observed in non-normative CA, *F*_(2,116)_ = 4.70, *p* = 0.011, among FCP supporters; and 14%, *F*_(2,98)_ = 7.85, *p* ≤ 0.001, among SLB supporters. Although we also found the expected path from SDO-D to non-normative CA through social competition intentions, no significant indirect effect is observed among FCP supporters, *b* = 0.05 *SE* = 0.04, 95% CI [−0.018, 0.153], a *post-hoc* power analysis showed a power (1 – β) of 0.47; or among SLB supporters, *b* = 0.06 *SE* = 0.04, 95% CI [−0.005, 0.149], power (1 – β) of 0.36.

##### SDO-E on Normative CA Through Social Competition

We also observe that SDO-E is not a significant predictor of social competition neither among FCP supporters nor among SLB supporters (see Figure 2). Nevertheless, social competition predicts normative CA, among both FCP supporters and SLB supporters. The model explains 19% of the variability observed in normative CA, *F*_(2,116)_ = 13.56, *p* ≤ 0.001, among FCP supporters; and 26%, *F*_(2,98)_ = 17.04, *p* ≤ 0.001, among SLB supporters. No significant indirect effect is observed among FCP supporters, *b* = 0.09 *SE* = 0.07, 95% CI [−0.053, 0.214], a *post-hoc* power analysis showed a power (1 – β) of 0.26; or among SLB supporters, *b* = 0.15 *SE* = 0.12, 95% CI [−0.058, 0.419], power (1 – β) of 0.33.

##### SDO-E on Non-normative CA Through Social Competition

We also observed that SDO-E maintains a positive and significant direct effect on non-Normative CA even after the mediator was included in the equation (see Figure 2), among both FCP supporters, *b* = 0.29, *p* ≤ 0.001 and SLB supporters, *b* = 0.25, *p* = 0.020. Social competition also significantly predicted non-normative CA, among both FCP supporters and SLB supporters. The model explains 15% of the variability observed in non-normative CA, *F*_(2,116)_ = 10.23, *p* ≤ 0.001, among FCP supporters; and 14%, *F*_(2,98)_ = 8.10, *p* ≤ 0.001, among SLB supporters. No indirect effect of SDO-E on non-normative CA through social competition emerged among FCP supporters, *b* = 0.02 *SE* = 0.02, 95% CI [−0.012, 0.081], a *post-hoc* power analysis showed a power (1 – β) of 0.15; or among SLB supporters, *b* = 0.05 *SE* = 0.04, 95% CI [−0.021, 0.127], power (1 – β) of 0.24.

### Discussion

Results of Study 2 support our mediational hypothesis only with the SDO-D subdimension on normative CA. Specifically, among FCP supporters (low-status group pursuing the champion title), we observed a partial mediation of SDO-D on normative CA, since SDO-D has both a direct and indirect effect (through social competition intentions) on normative CA. Regarding non-normative CA, although we observed the expected path and the direct effect of SDO-D on non-normative CA disappeared after the mediator (social competition) was included, we observed no significant indirect effect.

Regarding the SDO-E dimension, we observed that, SDO-E was not a reliable predictor of social competition, among both FCP and SLB supporters. Moreover, among FCP supporters (low-status group), SDO-E had a positive direct effect on normative CA, but this effect disappeared after including the mediator in the regression equation. We also observed a positive direct effect of SDO-E on non-normative CA, which was virtually unchanged after the mediator (social competition) was accounted for. This suggests that SDO-E is a strong predictor of non-normative CA, among FCP supporters, and this relation is not explained through social competition intentions.

Among SLB supporters (high-status group), we observed that neither SDO-D nor SDO-E have direct or indirect effects on Normative CA. We also observed that both SDO-D and SDO-E had a positive direct effect on non-normative CA, which was slightly reduced after taking into account the mediator (social competition). This suggests that both SDO-D and SDO-E are strong predictors of non-normative CA among SLB supporters (the high-status group), and this relation is not explained through social competition intentions.

## General Discussion

Previous research has determined that SDO endorsement among members of low-status groups is associated with negative feelings about ingroup membership (e.g., Levin and Sidanius, 1999; Jost et al., 2004) or as a way to deal and cope with cognitive dissonance, anxiety, discomfort and uncertainty, resulting from their disadvantaged condition (e.g., Jost et al., 2004). By this view, SDO is expected to be negatively associated with willingness to engage in collective action, to favor the ingroup, among members of low-status groups (e.g., Jost et al., 2012, 2017; Osborne et al., 2018).

However, recent evidence (e.g., Caricati and Sollami, 2017; Owuamalam et al., 2017) suggests that support for hierarchical organized intergroup relations and group status differentials (i.e., SDO), among members of low-status groups, may represent a strategy to guarantee a possible future ingroup high-status. Specifically, we proposed that under competitive social structure conditions, SDO should boost collective action tendencies, among members of low-status groups, as a means to favor the ingroup by increasing members' motivation to engage in direct competition with the relevant high-status outgroup, reflecting an ideological strategy to guarantee the legitimacy of future ingroup status-enhancement within the prevailing hierarchical system.

We tested this idea with two studies under highly unstable social structure contexts (i.e., the necessary conditions for social competition strategies to emerge among members of low-status groups). In Study 1 we induced Low vs. High ingroup status with information from a University ranking and in Study 2 we used football team supporters from two rival teams, during a football championship.

We predicted that, in both studies, members of the lower-status groups to show stronger beliefs that the status groups' relative positions were unstable and illegitimate, higher motivation to favor the ingroup and/or to derogate the outgroup, as well as stronger motivation to engage in social competition, than would members of the higher-status groups. As expected, in both studies, participants who belong to groups in a lower status position reported higher beliefs in unstable and illegitimate intergroup status relations than participants from groups in a higher status position. In Study 1, as expected, participants in the Low-status condition showed higher motivation to derogate the outgroup and stronger social competition intentions, than participants in the high-status condition. However, in Study 2 there were no differences in these measures between members of higher and lower status groups. We may think that this may have occurred because the final ranking positions of the football teams and the championship winner (i.e., the group's statuses) were not yet established, as the championship was still ongoing, and hence both groups were equally motivated to compete for the title.

Also, as expected, we found SDO to be positively related to social competition intentions and collective action tendencies, among members of the low-status group. Moreover, consistent in both studies, SDO was associated with outgroup derogation but not with ingroup favoritism, among members of the low-status groups. A similar pattern emerged among members of the high-status groups, in line with data from previous research.

Finally, we expected a mediational process, among members of the low-status groups, such that SDO should boost collective action tendencies to favor the ingroup by increasing participants' motivation to engage in direct competition with the other relevant high-status outgroup. In Study 1, we found that, indeed, SDO increased participants' motivation to get involved in collective action, by boosting their motivation to engage in direct competition with the higher-status outgroup. In other words, participants' support for group-based hierarchies and inequality, and beliefs that social groups differ and should differ in value, seems to justify and to legitimate the engagement in direct competition (and subsequently in collective efforts), to reverse status positions and overcome the high-status outgroup, within the prevailing hierarchical system. In Study 2, we found a similar mediation pattern, but only with the SDO-D dimension and regarding normative collective actions. Although the mediation pattern for non-normative collective action was similar to this one, the indirect effect was not significant.

Thus, although, previous research has determined that SDO should be negatively related with collective action among members of low-status groups, our research shows that support for group-based hierarchy and inequality may represent an ideological strategy to guarantee the legitimacy of future ingroup status-enhancement, when changing intergroup status positions is a possibility (see Owuamalam et al., 2018, 2019). Indeed, overall, we found consistent positive associations (direct and/or indirect effects) between SDO and collective action, among members of the low-status group, suggesting that, indeed, this relationship is not always negative as previously research has determined.

### Theoretical Implications

We believe that our work has relevant implications for both SDT and collective action research. Our work stresses the importance of considering features of the social structure in SDO research, such as beliefs in the stability of status relations between groups. Indeed, in highly unstable contexts where social competition is highly encouraged, it seems that SDO may in fact boost collective action, among members of low-status groups, and not necessarily be opposed to ingroup's interests and undermine individuals' motivation to get involved in collective action, as previously suggested. Moreover, our work adds more evidence to SDO's conceptualization as a function of situational and contextual factors, acting to justify and legitimate individuals' attitudes and/or action (cf. Guimond et al., 2003). Thus, investigating the situational and contextual factors underlying individuals' adherence to hierarchy-enhancing ideologies, such as SDO, may contribute to understand the maintenance, normalization and perpetuation of social stratification and inequalities between social groups, such as, institutional or systemic racism.

Finally, our work also highlights the importance of including ideological processes on collective action research (see Jost et al., 2017; Choma et al., 2019; Mikołajczak and Becker, 2019). Specifically, SDO, as a cognitive and ideological justification for the maintenance of intergroup inequality, hierarchical intergroup relations, and status and power differentiation between groups, is particularly relevant to collective action research, since these actions are predominantly expected to be directed to decrease group differentiations and promote intergroup equality. Thus, including system-justifying or hierarchy-enhancing ideologies, such as SDO, in collective action research may contribute to better understand individuals' motivation to engage in, and the meanings of, social movements.

### Limitations and Future Directions

In spite of the potential contribution of our results, there are also potential limitations that should be addressed in future research.

We start by addressing the limitations regarding the sample size in Study 1. Indeed, sample sizes were *n* = 39 in the Low-status condition and *n* = 38 in the High-status condition, which can be considered very small for mediation analyses (e.g., Fritz and MacKinnon, 2007). Even though we detected the expected mediated effect, results should be interpreted with caution. Therefore, future research may seek to replicate this study with a larger sample, using results from an annual World University Ranking (e.g., Quacquarelli Symonds QS World University Rankings; Center for World University Rankings – CWUR), which can easily be analyzed by country, allowing to be applied with students from any University, and even use a cross-group design.

Regarding Study 2, some limitations should also be addressed. We found consistent full mediations in Study 1, and partial mediations and direct effects in Study 2, among members of low-status groups. It is possible that the results in Study 2 may have been influenced by the unique characteristics of football context, such as, the strong emotional commitment that football invokes (see Shakina et al., 2020); the impact of participating in ritual gatherings (e.g., matches), of the symbols representative of the group (e.g., flags), and emotional entrainment, on identification and commitment with the group (Von Scheve et al., 2014). It may also have been influenced by other variables not included or controlled in the study, such as, the degree/intensity of rivalry between groups (being extremely intense between football teams, more than between Universities) that shape identities, attitudes and behaviors (e.g., Benkwitz and Molnar, 2012); degree of fan loyalty which reflects a persistent attitude resistant to change, and creates biases in cognitive processing and provides a guide to behavior (Funk and James, 2001); fanographics (i.e., variables that measure fans' relationship with the sport or club; e.g., time dedicated to the team; de Carvalho et al., 2015).

Moreover, in Study 1, status positions were already stablished for the current year, and no individual or collective action could change that result at least in the short-term. In Study 2, although teams' positions were based on the teams' scores at the time our study was conducted and, obviously, results from the previous championship (that were consistent with current scores at the time) were prominent in the minds of the participants, ranking (or status) positions were not yet defined or established and therefore, could reverse. In other words, “everything was at stake” for both teams. Indeed, at the end of the championship (2019/2020), teams' positions reversed and the FCP overcame the former champion (SLB). This shows the degree of instability and uncertainty teams' supporters may have experienced. It would have been interesting to have replicated the study and observe the patterns of association between our measures after the championship ended. Future research may consider this longitudinal procedure.

Moreover, interestingly, as discussed above, although SDO-D is expected to be more related, than SDO-E, to support for hostile and aggressive attitudes and behaviors especially under intergroup competition or conflict (Ho et al., 2012, 2015), the direct effect of SDO-D on non-normative collective action (Study 2) was weaker, compared to normative collective action, and disappeared after including the mediator. Surprisingly, SDO-E, that typically is not related with violent or overt confrontation and believed to be more subtle in nature (Ho et al., 2015), showed a stronger direct effect that remained unchanged after including the mediator. Moreover, in this context, and contrasting with Study 1, SDO-E was not related with social competition. As stressed by Ho et al. (2012), although previous research suggests that SDO-D and SDO-E are related to distinct intergroup phenomena, results are still inconclusive. For instance, Ho et al. (2015) found inconsistent results among Black participants in the relationship between SDO-D and SDO-E with criterion variables, suggesting that there may be moderators that may help to better understand how SDO-D and SDO-E relates with some intergroup phenomena. Moreover, research considering these two dimensions (i.e., the SDO_7_ scale; Ho et al., 2015), and, in particular, under contexts of social competition, it is still scarce. Thus, more research is needed to better understand the meaning of, and motivations underlying, each SDO subdimension, taking into account social status and the existing (or perceived) social structure, and in particular, under competition-based and conflict-based settings.

We also found, consistently in both studies, that SDO was positively related with only outgroup derogation, but not with ingroup favoritism, among members of low-status groups. Indeed, under such competition-based and conflict-based contexts, the need for intergroup distinctiveness provides a fertile ground for conflict and hate, leading to great hostility toward the outgroup (e.g., Tajfel, 1978; Brewer, 1999). Moreover, individual's motivation to get involved in social competition and intergroup conflict “can be a result of a cooperative desire to help the ingroup (“ingroup love”), an aggressive/competitive motivation to hurt the outgroup or increase the gap between the groups (“outgroup hate”), or a combination of both” (Weisel and Böhm, 2015, p. 110). Thus, future research should address these aspects to better understand the relations between SDO and outgroup derogation (and ingroup favoritism), among low-status groups under competition-based and conflict-based contexts.

## Data Availability Statement

The data supporting the conclusions of this article is available at https://osf.io/b9xf8/.

## Author Contributions

CC and IP contributed to conception and design of the studies. CC conducted data collection, organized database, performed the statistical analysis, and wrote the first draft of the manuscript with support from IP. All authors contributed to results interpretation, to preparation and presentation of the final manuscript with critical review, commentary, and revision in different stages of the manuscript. All authors read and approved the submitted version.

## Conflict of Interest

The authors declare that the research was conducted in the absence of any commercial or financial relationships that could be construed as a potential conflict of interest.

## Footnotes

^1^The scale was translated from English to Portuguese by one of the researchers and by an experienced translator. The two translations were compared and discussed, and some adjustments were made together with a third experienced researcher.

^2^The full scale included another 4 items of collective initiatives against/to contest the current University's position in the Ranking. An initial principal components factorial analysis conducted on the full scale confirmed the existence of two distinct factors accounting for 80% of the total variance. Thus, initially, we averaged the scores of the items 1–6 to a *collective action* index (Cronbach's α = 0.93), and the items 7–10 to a *contestation* index (Cronbach's α = 0.95). However, since contestation presented similar patterns of results to those found with collective action, henceforth, we only discuss the results with the collective action measure. Means, standard deviations, and correlations between measures by status condition, including contestation measure, are available as online supplementary materials (OSM) 1 at https://osf.io/b9xf8/

^3^Mediation analysis with contestation presents the same pattern. Results and figures of the mediation models with contestation are available at the OSM 2. We only found a different pattern with the SDO-D dimension in the High-status condition: the indirect effect of SDO-D on contestation through social competition is positive and significant, *b* = 0.38 *SE* = 0.18, 95% CI [0.065, 0.799], contrasting with the indirect effect of SDO-D on collective action that was non-significant. This may reflect a desire to increase the differences between the two Universities, or simply a desire of enhancing ingroup position in the global ranking (i.e., they are already in 1st place in the national ranking and want to rise their position in the world ranking).

^4^We calculated statistical power using Monte Carlo simulations with Schoemann et al.'s (2017) online calculator: https://schoemanna.shinyapps.io/mc_power_med/, following authors' recommendations.

^5^We discarded item 2 because it presented low communality (<0.20) in a preliminary analysis.

## References

[B1] Benkwitz A. Molnar G. (2012). Interpreting and exploring football fan rivalries: an overview. Soccer Soc. 13, 479–494. 10.1080/14660970.2012.677224

[B2] Blanz M. Mummendey A. Mielke R. Klink A. (1998). Responding to negative social identity: a taxonomy of identity management strategies. Eur. J. Soc. Psychol. 28, 697–729. 10.1002/(SICI)1099-0992(199809/10)28:5<697::AID-EJSP889>3.0.CO;2-#

[B3] Brandt M. J. Reyna C. (2017). Individual differences in the resistance to social change and acceptance of inequality predict system legitimacy differently depending on the social structure. Eur. J. Person. 31, 266–278. 10.1002/per.2100 28706346 PMC5485172

[B4] Brewer M. B. (1999). The psychology of prejudice: ingroup love and outgroup hate? J. Soc. Iss. 55, 429–444. 10.1111/0022-4537.00126

[B5] Caricati L. Sollami A. (2017). Perceived legitimacy follows in-group interests: evidence from intermediate-status groups. Br. J. Soc. Psychol. 56, 197–206. 10.1111/bjso.12174 27861996

[B6] Caricati L. Sollami A. (2018). Contrasting explanations for status-legitimacy effects based on system justification theory and social identity theory. J. Theor. Soc. Psychol. 2, 13–25. 10.1002/jts5.15

[B7] Choma B. Hodson G. Jagayat A. Hoffarth M. R. (2019). Right-wing ideology as a predictor of collective action: a test across four political issue domains. Polit. Psychol. 41, 303–322. 10.1111/pops.12615

[B8] de Carvalho M. Boen F. Sarmento J. P. Scheerder J. (2015). What brings youngsters into the stadium? Sociopsychological predictors of soccer attendance among Belgian and Portuguese young fans. Revista Portuguesa de Ciências Do Desporto 15, 21–40. 10.5628/rpcd.15.01.21

[B9] Duckitt J. Sibley C. (2009). A dual-process motivational model of ideology, politics, and prejudice. Psychol. Inq. 20, 98–109. 10.1080/1047840090302854026391187

[B10] Fritz M. S. MacKinnon D. P. (2007). Required sample size to detect the mediated effect. Psychol. Sci. 18, 233–239. 10.1111/j.1467-9280.2007.01882.x 17444920 PMC2843527

[B11] Funk D. James J. (2001). The psychological continuum model: a conceptual framework for understanding an individual's psychological connection to sport. Sport Manage. Rev. 4, 119–150. 10.1016/S1441-3523(01)70072-1

[B12] Guimond S. Dambrun M. Michinov N. Duarte S. (2003). Does social dominance generate prejudice? Integrating individual and contextual determinants of intergroup cognitions. J. Person. Soc. Psychol. 84, 697–721. 10.1037/0022-3514.84.4.697 12703644

[B13] Hayes A. F. (2018). Introduction to Mediation, Moderation, and Conditional Process Analysis: A Regression-Based Approach, 2nd Edn. New York, NY: The Guilford Press.

[B14] Ho A. K. Sidanius J. Kteily N. Sheehy-Skeffington J. Pratto F. Henkel K. E.. (2015). The nature of social dominance orientation: theorizing and measuring preferences for intergroup inequality using the new SDO7 scale. J. Pers. Soc. Psychol. 109, 1003–1028. 10.1037/pspi0000033 26479362

[B15] Ho A. K. Sidanius J. Pratto F. Levin S. Thomsen L. Kteily N.. (2012). Social dominance orientation: revisiting the structure and function of a variable predicting social and political attitudes. Pers. Soc. Psychol. Bull. 38, 583–606. 10.1177/0146167211432765 22215697

[B16] Jetten J. Iyer A. (2010). Different meanings of the social dominance orientation concept: predicting political attitudes over time. Br. J. Soc. Psychol. 49, 385–404. 10.1348/014466609X435723 19397844

[B17] Jost J. T. Banaji M. R. Nosek B. A. (2004). A decade of system justification theory: accumulated evidence of conscious and unconscious bolstering of the status quo. Polit. Psychol. 25, 881–919. 10.1111/j.1467-9221.2004.00402.x

[B18] Jost J. T. Becker J. Osborne D. Badaan V. (2017). Missing in (collective) action: ideology, system justification, and the motivational antecedents of two types of protest behavior. Curr. Direct. Psychol. Sci. 26, 99–108. 10.1177/0963721417690633

[B19] Jost J. T. Chaikalis-Petritsis V. Abrams D. Sidanius J. Van Der Toorn J. Bratt C. (2012). Why men (and women) do and don't rebel effects of system justification on willingness to protest. Pers. Soc. Psychol. Bull. 38, 197–208. 10.1177/0146167211422544 21911420

[B20] Jost J. T. Thompson E. P. (2000). Group-based dominance and opposition to equality as independent predictors of self-esteem, ethnocentrism, and social policy attitudes among African Americans and European Americans. J. Exp. Soc. Psychol. 36, 209–232. 10.1006/jesp.1999.1403

[B21] Kugler M. B. Cooper J. Nosek B. A. (2010). Group-based dominance and opposition to equality correspond to different psychological motives. Soc. Justice Res. 23, 117–155. 10.1007/s11211-010-0112-5

[B22] Leach C. W. van Zomeren M. Zebel S. Vliek M. L. W. Pennekamp S. F. Doosje B.. (2008). Group-level self-definition and self-investment: a hierarchical (multicomponent) model of in-group identification. J. Pers. Soc. Psychol. 95, 144–165. 10.1037/0022-3514.95.1.144 18605857

[B23] Lehmiller J. J. Schmitt M. T. (2007). Group domination and inequality in context: evidence for the unstable meanings of social dominance and authoritarianism. Eur. J. Soc. Psychol. 37, 704–724. 10.1002/ejsp.383

[B24] Levin S. (2004). Perceived group status differences and the effects of gender, ethnicity, and religion on social dominance orientation. Polit. Psychol. 25, 31–48. 10.1111/j.1467-9221.2004.00355.x

[B25] Levin S. Federico C. M. Sidanius J. Rabinowitz J. L. (2002). Social dominance orientation and intergroup bias: the legitimation of favoritism for high-status groups. Pers. Soc. Psychol. Bull. 28, 144–157. 10.1177/0146167202282002

[B26] Levin S. Sidanius J. (1999). Social dominance and social identity in the United States and Israel: ingroup favoritism or outgroup derogation? Polit. Psychol. 20, 99–126. 10.1111/0162-895X.0013825652084

[B27] Liu J. H. Huang L. L. McFedries C. (2008). Cross-sectional and longitudinal differences in social dominance orientation and right-wing authoritarianism as a function of political power and societal change. Asian J. Soc. Psychol. 11, 116–126. 10.1111/j.1467-839X.2008.00249.x

[B28] Mikołajczak G. Becker J. C. (2019). What is (un) fair? political ideology and collective action. J. Soc. Polit. Psychol. 7, 810–829. 10.5964/jspp.v7i2.1230

[B29] Morrison K. K. Fast N. J. Ybarra O. (2009). Group status, perceptions of threat, and support for social inequality. J. Exp. Soc. Psychol. 45, 204–210. 10.1016/j.jesp.2008.09.004

[B30] Mummendey A. Klink A. Mielke R. Wenzel M. Blanz M. (1999). Socio-structural characteristics of intergroup relations and identity management strategies: results from a field study in East Germany. Eur. J. Soc. Psychol. 29, 259–285. 10.1002/(SICI)1099-0992(199903/05)29:2/3<259::AID-EJSP927>3.0.CO;2-F

[B31] Osborne D. Jost J. T. Becker J. C. Badaan V. Sibley C. G. (2018). Protesting to challenge or defend the system? A system justification perspective on collective action. Eur. J. Soc. Psychol. 49, 244–269. 10.1002/ejsp.252226914824

[B32] Owuamalam C. K. Rubin M. Issmer C. (2016). Reactions to group devaluation and social inequality: a comparison of social identity and system justification predictions. Cogent Psychol. 3, 1–29. 10.1080/23311908.2016.1188442

[B33] Owuamalam C. K. Rubin M. Spears R. (2018). Addressing evidential and theoretical inconsistencies in system-justification theory with a social identity model of system attitudes. Curr. Direct. Psychol. Sci. 27, 91–96. 10.1177/0963721417737136

[B34] Owuamalam C. K. Rubin M. Spears R. (2019). Revisiting 25 years of system motivation explanation for system justification from the perspective of social identity model of system attitudes. Br. J. Soc. Psychol. 58, 362–381. 10.1111/bjso.12285 30328122

[B35] Owuamalam C. K. Rubin M. Spears R. Weerabangsa M. M. A. (2017). Why do people from low-status groups support class systems that disadvantage them? a test of two mainstream explanations in Malaysia and Australia. J. Soc. Iss. 73, 80–98. 10.1111/josi.12205

[B36] Perry R. Sibley C. G. Duckitt J. (2013). Dangerous and competitive worldviews: a meta-analysis of their associations with social dominance orientation and right-wing authoritarianism. J. Res. Pers. 47, 116–127. 10.1016/j.jrp.2012.10.004

[B37] Pinto I. R. Marques J. M. Levine J. M. Abrams D. (2016). Membership role and subjective group dynamics: impact on evaluative intragroup differentiation and commitment to prescriptive norms. Group Proc. Intergroup Relat. 19, 570–590. 10.1177/1368430216638531

[B38] Pratto F. Sidanius J. Stallworth L. M. Malle B. F. (1994). Social dominance orientation: a personality variable predicting social and political attitudes. J. Pers. Soc. Psychol. 67, 741–763. 10.1037/0022-3514.67.4.741

[B39] Reynolds K. J. Turner J. C. Haslam S. A. Ryan M. K. (2001). The role of personality and group factors in explaining prejudice. J. Exp. Soc. Psychol. 37, 427–434. 10.1006/jesp.2000.1473

[B40] Rubin M. Badea C. Jetten J. (2014). Low status groups show in-group favoritism to compensate for their low status and compete for higher status. Group Proc. Intergroup Relat. 17, 563–576. 10.1177/1368430213514122

[B41] Schmitt M. T. Branscombe N. R. Kappen D. M. (2003). Attitudes toward group-based inequality: social dominance or social identity? Br. J. Soc. Psychol. 42, 161–186. 10.1348/014466603322127166 12869240

[B42] Schoemann A. M. Boulton A. J. Short S. D. (2017). Determining power and sample size for simple and complex mediation models. Soc. Psychol. Person. Sci. 8, 379–386. 10.1177/1948550617715068

[B43] Shakina E. Gasparetto T. Barajas A. (2020). Football fans' emotions: uncertainty against brand perception. Front. Psychol. 11:659. 10.3389/fpsyg.2020.00659 32499734 PMC7242735

[B44] Sibley C. G. Wilson M. S. (2007). Political attitudes and the ideology of equality: differentiating support for liberal and conservative political parties in New Zealand. N Z. J. Psychol. 36, 72–84.

[B45] Sidanius J. Cotterill S. Sheehy-Skeffington J. Kteily N. Carvacho H. (2017). Social dominance theory: explorations in the psychology of oppression, in The Cambridge Handbook of the Psychology of Prejudice, eds Sibley C. G. Barlow F. K. (Cambridge: Cambridge University Press), 149–187. 10.1017/9781316161579.008

[B46] Sidanius J. Pratto F. Mitchell M. (1994). In-group identification, social dominance orientation, and differential intergroup social allocation. J. Soc. Psychol. 134, 151–167. 10.1080/00224545.1994.9711378

[B47] Tajfel H. (1978). Differentiation *B*etween *S*ocial *Groups: Studies in the Social Psychology of Intergroup Relations*. London: Academic Press.

[B48] Tajfel H. Turner J. C. (1979). An integrative theory of intergroup conflict, in The Social Psychology of Intergroup Relations, eds Austin W. G. Worchel S. (New York: Brooks/Cole), 33–47.

[B49] Von Scheve C. Beyer M. Ismer S. Kozłowska M. Morawetz C. (2014). Emotional entrainment, national symbols, and identification: a naturalistic study around the men's football World Cup. Curr. Sociol. 62, 3–23. 10.1177/0011392113507463

[B50] Weisel O. Böhm R. (2015). “Ingroup love” and “outgroup hate” in intergroup conflict between natural groups. J. Exp. Soc. Psychol. 60, 110–120. 10.1016/j.jesp.2015.04.008 26339099 PMC4518042

